# Dendritic Cell-Targeted Vaccines

**DOI:** 10.3389/fimmu.2014.00255

**Published:** 2014-05-30

**Authors:** Lillian Cohn, Lélia Delamarre

**Affiliations:** ^1^Laboratory of Molecular Immunology, Rockefeller University, New York, NY, USA; ^2^Genentech, South San Francisco, CA, USA

**Keywords:** dendritic cells, MHC class I, CD8^+^ T cells, vaccination, adjuvants, immunologic

## Abstract

Despite significant effort, the development of effective vaccines inducing strong and durable T-cell responses against intracellular pathogens and cancer cells has remained a challenge. The initiation of effector CD8^+^ T-cell responses requires the presentation of peptides derived from internalized antigen on class I major histocompatibility complex molecules by dendritic cells (DCs) in a process called cross-presentation. A current strategy to enhance the effectiveness of vaccination is to deliver antigens directly to DCs. This is done via selective targeting of antigen using monoclonal antibodies directed against endocytic receptors on the surface of the DCs. In this review, we will discuss considerations relevant to the design of such vaccines: the existence of DC subsets with specialized functions, the impact of the antigen intracellular trafficking on cross-presentation, and the influence of maturation signals received by DCs on the outcome of the immune response.

## Introduction

Vaccination is the most effective way to prevent the spread of infectious diseases. We classify vaccines into two main types: preventative or therapeutic. Preventative vaccines typically elicit generation of specific antibodies and memory B cells. They are designed to block the spread of infection through these humoral immune responses (1). Alternatively, therapeutic vaccines are designed as a treatment to eradicate the cause of disease. Therapeutic vaccines are typically intended to activate or induce cytotoxic antigen-specific CD8^+^ T cells to eliminate virally infected cells or cancer cells. There are many conditions for which vaccination has diminished the devastating effects of disease, and the discovery of these vaccines has largely resulted from successful trial and error. However, there are many diseases for which no vaccine exists; e.g., human immunodeficiency virus, hepatitis C, malaria, and cancer. It is likely that cytotoxic CD8^+^ T-cell activity will be required to protect patients from these chronic conditions. For this reason, efforts are required to develop carefully designed therapeutic vaccines that will derive from our increasing understanding behind the mechanisms of the human immune system. Dendritic cells (DCs) are the antigen-presenting cells that initiate and direct adaptive immune responses, and thus are critically important in our consideration of vaccines designed to induce cellular immunity.

DCs induce and regulate immunity against pathogens, and tolerance against self-antigens and commensal microorganisms (2–4). In their immature state, DCs reside in the periphery where they are situated to recognize and capture antigens. Upon receiving an activating stimulus, DCs migrate to lymphoid organs whereby they present processed peptides derived from captured antigens to T cells in the context of major histocompatibility complex (MHC) class I or II (5). The immune response initiated by the DCs is dependent upon the context in which the antigen was captured. DCs induce tolerance under steady-state conditions, in the absence of infection or inflammation – generally in this case it is self-antigens that are processed and presented. The exact nature and state of tolerogenic DCs remain elusive. However, there is an increasing body of evidence suggesting that microenvironmental signals condition DCs to become tolerogenic (6). In this process, beta-catenin activation appears to play a central role (7–10), although other mechanisms also contribute to tolerance induction (8). In the presence of inflammatory signals, such as microbial products, proinflammatory cytokines, and other endogenous signals, DCs undergo a process called maturation. DC maturation is associated with dramatic functional and morphological changes that lead to an optimized ability to initiate T-cell immunity. It is characterized by an increase in cell surface expression of MHCI and MHCII molecules and accessory/costimulatory molecules, increased antigen processing, and induction of specific cytokine production (5). Maturation depends on both the nature of the stimuli and its extent and combination (11). Additionally, the DC compartment is diverse and contains different cell types with both conserved and unique functions and specialties. Indeed, different DC subsets possess different capacity for antigen presentation, cytokine production, and microbial sensing (12). Thus, it seems that different types of immune responses are initiated by specialized DC subsets.

The critical role of DCs to activate CD8^+^ T cells makes them an attractive target for vaccination against intracellular pathogens and diseases for which cellular immunity seems to be a crucial part of the immune response. One approach is cell-based immunotherapy with *ex vivo* generated DCs loaded with antigens (13). This approach however is laborious and expensive, and thus far clinical results have been limited. Another more promising approach to direct DCs involves selective targeting to DC-specific endocytic receptors by monoclonal antibody coupled or fused to a desired antigen. These complexes are internalized by the DCs, trafficked through the intracellular vesicular system, processed, and the antigenic peptides are loaded onto MHC and presented to T cells (14, 15). In mice, in the presence of adjuvant, these antigen–antibody conjugates induce robust immune responses (16). However, in the absence of adjuvant, these conjugates can promote a tolerogenic state (17). This *in situ* targeting strategy is in its infancy in human patients. The first clinical trials to evaluate this vaccine approach are in progress and their preliminary results are encouraging (18–20). Recent progress in understanding the biology of DCs should further help with optimization of a DC-targeted vaccine strategy: (1) identification of the human DC subsets with superior capacity at initiating CD8^+^ T-cell responses if any, (2) selection of the receptors based on expression pattern to target the desired DC subset(s), and also their ability to deliver antigen to intracellular compartments for processing and loading on MHC and (3) choice of the adjuvant(s) to induce the desired immune response. In this review, we will discuss the issues relevant to human vaccination through *in vivo* DC targeting: the existence of multiple DC subsets with specialized functions, how DCs handle external antigen for presentation on MHCI and the intracellular targeting that induces optimal immune responses, and finally the role of DC maturation signals in orchestrating the immune outcome.

## Dendritic Cell Subsets

Increasingly it has become apparent that there exists a division of labor among DC subsets in both mice and in humans (12, 21, 22). The number of DC subsets identified, and the functional studies performed both *in vivo* in mice and *in vitro* using isolated DC subsets from humans yield evidence for specialization in T-cell priming and induction of immune responses, although the functions of the different DC subsets can partially overlap.

While the mouse DC network has been quite well characterized, until recently thorough studies with human blood DCs have been difficult due to their paucity in the blood and the difficulty to access human tissues. However recent genome-wide expression profiling studies helped identify the potential human counterparts to the mouse DC subsets (23, 24).

**Figure 1:**
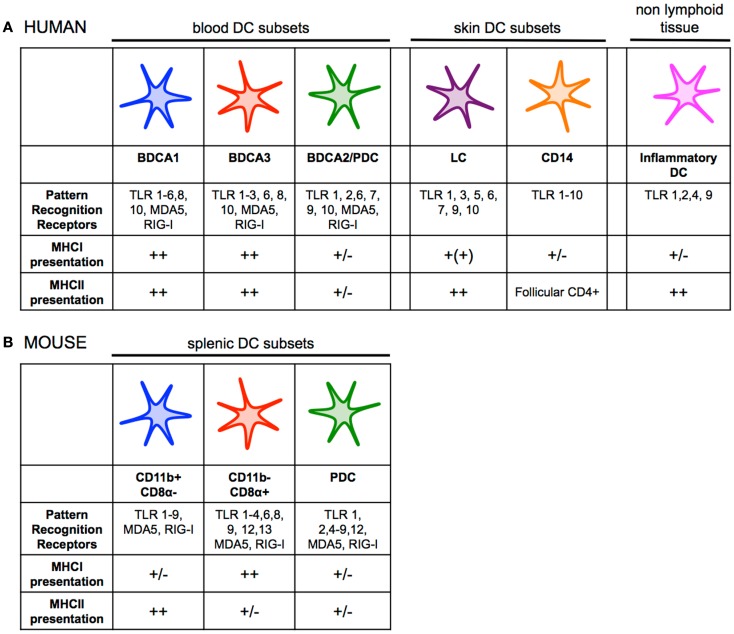
**(A)** Human dendritic cell subsets have overlapping functions and phenotypes, but also show some degree of specialization. BDCA1^+^ DCs and BDCA3^+^ DCs both efficiently present antigen on MHCI and MHCII. pDCs can present antigen to CD4^+^ and CD8^+^ T cells, but likely their primary role in the immune response is the production of type I interferon during viral infection. LCs seem to be specialized for cross-presentation on MHCI, while CD14^+^ dermal DCs prime naïve CD4^+^ T cells to generate follicular helper T cells. Inflammatory DCs are monocyte-derived, and are present at sites of inflammation. There is also partial overlap between expression of PRRs among DC subsets. **(B)** A clear division of labor exists among mouse splenic dendritic cell subsets. CD11b^−^ CD8α^+^ DCs are far superior and essential at priming CD8^+^ T-cell responses, while CD11b^+^ DCs are specialized for presenting antigen on MHCII to stimulate helper T-cell immunity. pDCs can present antigen to CD4^+^ and CD8^+^ T cells, but likely their primary role in the immune response is the production of type I interferon during viral infection like their human counterparts. There is overlap between expression of PRRs among DC subsets, although CD11b^−^ CD8α^+^ DCs express much higher levels of TLR3 while CD11b^+^ DCs uniquely express TLR5 and TLR7 (30, 35, 41, 58, 64, 147–151).

Human and mouse DCs can be divided in two main subsets: plasmacytoid DCs (pDCs) and conventional/myeloid DCs (mDCs) (Figure 1). pDCs play a crucial role against viral infection by producing vast amounts of type I interferon in response toll-like receptors (TLR) 7 and 9 and intracellular sensor triggering (25). pDCs have been shown to be rather poor at antigen presentation in comparison to mDCs (26–28), although recent studies suggest that efficient antigen delivery to pDCs via endocytic receptors can lead to robust presentation on both MHCI and MHCII (29–31). However, the influence of antigen presentation by pDCs *in vivo* has yet to be understood. Additionally, in mice there is evidence that suggest pDCs play a major role in the generation of tolerance (32, 33). Whether this is true for human pDCs is still unknown.

Human mDCs can be divided into two main subsets based on the surface markers BDCA1/CD1c or BDCA3/CD141. A transcriptional comparison of mDCs has shown genetic similarity between human BDCA1^+^ DCs and BDCA3^+^ DCs from various tissues to murine CD11b^+^ and CD11b^−^ DCs, respectively (23, 34–36). Human BDCA3^+^ DCs express a number of markers unique to mouse CD11b^−^ CD8α^+^ and CD11b^−^ CD103^+^ DCs including the lectin receptor Clec9A/DNGR1, the chemokine receptor XCR1, and Necl2 (37–39). Further, human BDCA3^+^ DCs and mouse CD11b^−^ CD8α^+^ DCs share the expression of the transcription factors IRF8, BATF3 essential for their development (35, 40–43). Conversely, the transcriptional programing of mouse CD11b^+^ CD8α^−^ DCs and human BDCA1^+^ is dependent on IRF4 (44, 45). Functional studies of the mouse and human mDCs revealed differences between the two species, however. A clear division of labor exists among the two mDC subsets in mice with CD11b^−^ CD8α^+^ DCs and CD11b^−^ CD103^+^ DCs being far superior and essential at priming CD8^+^ T-cell responses, while CD11b^+^ CD8α^−^ DCs are specialized for presenting antigen on MHCII to stimulate helper T-cell immunity (12, 46, 47). This division of labor does not appear as clear between BDCA3^+^ DCs and BDCA1^+^ DCs at least in *in vitro* studies. Indeed both BDCA1^+^ DCs and BDCA3^+^ DCs can effectively cross-present antigens on MHCI (28, 31, 37, 38, 40, 41, 48–52). In addition, BDCA1^+^ DCs also produce high levels of IL-12 upon stimulation, a cytokine essential to inducing Th1 response and cross-priming of CD8^+^ T cells (28, 44, 48, 53, 54). BDCA3^+^ DCs and BDCA1^+^ DCs also exhibit a comparable capacity to present antigen on MHCII (28, 31). The skin contains two additional DC subsets that have been functionally characterized, the Langerhans cells (LCs) and the CD14^+^ DCs (36, 55). CD14^+^ DCs appear specialized in initiating humoral immune responses, while *in vitro*-derived LCs cross-present antigen on MHCI and prime CD8^+^ T cells of higher avidity as compared to CD14^+^ dermal DCs *in vitro* (26, 55). A side-by-side comparison of *in vitro*-derived LCs with CD14^+^ DCs suggests the two DC subset have similar capacity for cross-presentation (36). Importantly, LCs isolated from skin are incapable of cross-presentation of captured antigen, while they can present antigen on MHCII to CD4^+^ T cells (36, 56). Whether this deficiency is the result of the isolation procedure or a true characteristic of LCs remains to be confirmed.

Finally, the human equivalent of mouse inflammatory DCs was recently identified (57, 58). This DC subset is found in inflammatory microenvironments and can be divided into two main populations: CD16^+^ BDCA1^+^ DCs or CD16^−^ BDCA1^+^ DCs. They have characteristic gene patterns similar to that of DCs and macrophages, and thus are likely derived from monocytes. Although there are limited data on the functional specialization of human inflammatory DCs, they appear highly plastic like their murine counterparts (57, 58).

One limitation of the studies aimed at characterizing the functional capacity of human DCs is that they are performed *in vitro* using T-cell lines or memory T cells. These assays permit to evaluate the DCs’ capacity for antigen presentation. However, other factors are also important for DC function *in vivo* and priming of immune responses. The enhanced capacity of LCs to prime CD8^+^ T-cell responses may at least partially result from their ability to express IL-15 upon maturation (59, 60). The costimulatory molecule CD70 also promotes the priming of CD8^+^ T-cell responses and the generation of CD8^+^ T-cell memory (61–63). CD70 has been found to be expressed on LCs and all three blood DCs subsets upon maturation [(64, 65); Delamarre, personal communication]. Finally, DC function may depend on environmental cues, resident BDCA3^+^ DCs constitutively produce IL-10, possibly in a vitamin D3-dependent manner, and thus mediate T-cell tolerance rather than immunity at steady-state (66). Granulocyte–macrophage colony stimulating factor (GMCSF) has recently been shown to enhance the cross-presentation capacity of mouse CD11b^−^ CD8α^+^ DCs (67, 68).

Based on our current knowledge, there is no strong rational for the targeting of one DC subset over another to prime CD8^+^ T-cell responses in humans. Further *in vivo* studies are needed to identify the DC subsets if any that are specialized in cross-priming of CD8^+^ T cells. In this effort, it would be useful to better characterize DC subsets in non-human primates which appear to possess subpopulations of DCs that are similar to those present in humans (69) and therefore would be a more relevant model to humans than mice. Additionally, engagement of multiple DC subsets has been suggested to be important in generating a broad and potent T-cell response (70). For this reason, it may make sense to target a broad spectrum of DC subsets rather than a single DC subset.

## Antigen Cross-Presentation Pathways

In the design of rational DC-targeted vaccines, there are important considerations related to the delivery of antigen to DCs and the downstream processing of antigen by DCs. Delivery of antigen to DCs is essential to generate strong and prolonged T-cell responses. DCs are able to non-specifically phagocytose and macropinocytose pathogen-associated antigen and can also uptake antigen more specifically via lectin receptors, Fcγ receptors, and scavenger receptors (5). It has been shown that antigens can be efficiently targeted to DCs using antibodies against these endocytic receptors (15, 71). This takes advantage of antibodies against DC-specific endocytic receptors either coupled or fused to antigen or attached to nanoparticles containing antigen. In mice, this delivery method is hundreds of times more efficient and potent than untargeted antigens and offers options for antigen presentation on both MHCI and MHCII to CD8^+^ and CD4^+^ T cells, respectively (72). In addition, this strategy can also extend antigen cross-presentation to pDCs, which display poor phagocytosis and macropinocytosis capacity, and thus could potentially further promote T-cell responses *in vivo* (28–31). Another benefit of employing this strategy for antigen delivery is that it can allow for delivery to both immature and mature DCs. Unlike the non-specific phagocytosis and macropinocytosis, endocytic receptor-driven uptake continues even after DC maturation (73, 74). It would be best to selectively target DCs to reduce the dose of antigen required, while additionally limiting cross-presentation by other cell types. Indeed B cells and other non-hematopoietic cells can cross-present exogenous antigens, albeit with less efficiency than DCs, and induce peripheral tolerance under steady-state conditions and could potentially negatively impact vaccination efficacy (75–78). In addition, the binding of a target receptor by non-DCs may trigger a signaling pathway and thus may potentially have unwanted side effects.

DC subsets express different pattern of endocytic receptors and therefore the choice of receptor will determine which DC subsets are delivered antigen (Table 1). The choice of receptor also matters for other reasons. Some receptors can trigger DC maturation and induce immune responses of various natures as further discussed in the next section. In addition, they determine antigen intracellular trafficking that impacts antigen fate (28, 79). Some antibodies may also differentially alter antigen cross-presentation by modulating receptor trafficking (80). Antigen processing and loading on MHCI and MHCII happens in distinct intracellular compartments. For presentation on MHCII, antigen processing and loading occurs in the endosomal compartments, and peptide–MHCII complexes are transported to the plasma membrane (5).

**Table 1:** **Expression, intracellular localization, and ability to deliver antigen to MHCI and MHCII pathways of selected endocytic receptors and antigen**.

Receptors	Expression by DCs	Expression by other cells	Intracellular routing	DC activation	MHCI cross-presentation	MHCII presentation
CD11c	BDCA1^+^, BDCA3^+^, CD14^+^, LC, inflam. DC	Mono/MØ, neutrophil	Early endosome	No	+++ (Peptide)	?
CD32	BDCA1^+^, BDCA3^+^, CD14^+^, LC, inflam. DC, pDC	B, mono/MØ, NK, endothelial, neutrophil	Lysosome	Yes	+++ (Protein)	+++ (Protein)
CD40	BDCA1^+^, BDCA3^+^, CD14^+^, LC, inflam. DC, pDC	B, mono/MØ, endothelial	Early endosome	Yes	+++ (Peptide)	+++ (Peptide)
					+++ (Protein)	+++ (Protein)
CD205	BDCA1^+^, BDCA3^+^, CD14^+^, LC, inflam. DC, pDC	B, mono/MØ, T, endothelial	Lysosome	No	±(Peptide)	±(Peptide)
					+++ (Protein)	+++ (Protein)
CD206	BDCA1^+^, CD14^+^, inflam. DC	Mono/MØ, epithelial	Early endosome	No	+ (Peptide)	+++ (Protein)
					+++ (Protein)	
CD207	LC	–	Birbeck granules	No	−(Virus)	+++ (Protein)
						+++ (Virus)
CD209	CD14^+^, inflam. DC, pDC	Mono/MØ	Early endosome/lysosome	No	+++ (Protein)	+++ (Protein)
DNGR1	BDCA3^+^	–	Early endosome	No	+++ (Peptide)	+++ (Protein)
					+++ (Protein)	
Dectin-1	BDCA1^+^, CD14^+^	Mono/MØ	?	Yes	+++ (Protein)	?
DCIR	BDCA1^+^, LC, CD14^+^, pDC	B, mono/MØ	Early endosome/lysosome	No/suppressive?	+++ (Protein)	?

*Receptor selection for targeting DCs depends on four criteria: (1) whether the receptor is widely expressed among DC subsets, (2) whether other subsets of cells express the receptor, (3) upon internalization, where the receptor is trafficked, and finally (4) whether binding of this receptor activates DCs (28, 79, 80, 103, 105, 148, 149, 152–154). ?, not tested*.

**Figure 2:**
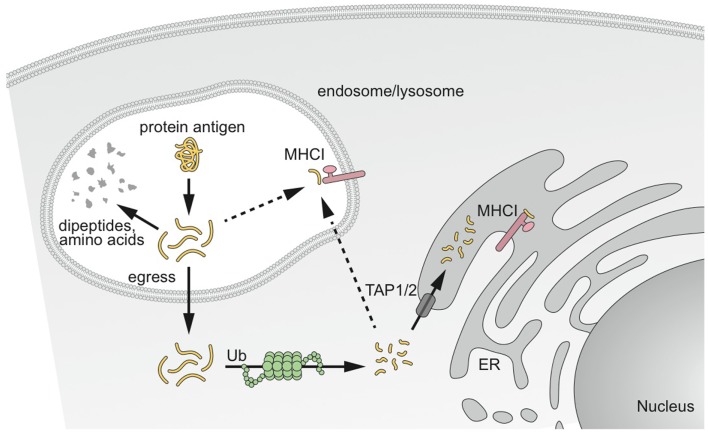
**MHCI cross-presentation pathways of captured antigens**. Antigen captured by DCs has different potential fates. Antigens destined for cross-presentation on MHCI have two different intracellular routes. Antigen can be transported from the endocytic vesicles to the cytosol to access the classical MHCI pathway involving proteasomal degradation and transport into the ER or back into the endosomal compartment for loading onto MHCI. The second pathway results in degradation and loading directly in endosomal compartments before peptide–MHCI complexes are transported to the plasma membrane. Modified from Delamarre and Mellman (14).

Two main intracellular pathways for the cross-presentation of exogenous antigen on MHCI have been reported. They are referred to as the “cytosolic” and “vacuolar” pathways (Figure 2) (81, 82).

From extensive work with human and mouse DCs, the “cytosolic pathway” appears the most predominant pathway. It is proteasome-dependent, and therefore requires that internalized proteins escape the intracellular trafficking pathway and access the cytosol, where they are processed by the proteasome and transported into the ER and possibly in endocytic compartments by TAP1/2 transporters for loading onto MHCI (83–85). The molecular mechanism underlying transport of antigen from endocytic compartments to cytosol remains largely unknown. No specific transporter has been identified yet, despite substantial efforts from different laboratories. A role of the ER-associated degradation (ERAD) machinery has been suggested in antigen export to the cytosol (86, 87). Consistent with this finding, the recruitment of ER-resident proteins to the phagosomes, via the ER molecule Sec22b, is required for cross-presentation (88). Regardless of the exact mechanism, antigen transfer to the cytosol is rate-limiting to antigen access to the MHCI pathway. When the antigen actively gains access to the cytosol using listeriolysin O or a fusogenic virus, cross-presentation is 10-fold more efficient (28). ISCOMATRIX adjuvant, a saponin-based adjuvant, which disrupts lysosomal membranes and facilitates antigen translocation to the cytosol also enhances antigen cross-presentation (89).

The “vacuolar pathway” is dependent upon lysosomal proteolysis by cathepsins and IRAP (90, 91) and independent of the proteasome and TAP1/2 transporters. Exogenous antigens are degraded directly in endocytic compartments by lysosomal proteases and trimmed for loading onto MHCI.

The reason why certain antigens are cross-presented by one pathway rather than the other is unknown. The nature and the form of the antigen, and the ability of the proteolytic environment to generate MHCI epitopes are certainly contributing factors (90). Maybe counter intuitively, antigen intracellular targeting does not appear to influence the intracellular-processing pathway for cross-presentation in human blood DCs as cross-presentation of antigen required proteasomal processing independently of its intracellular targeting (79).

A feature essential to the ability of DCs to efficiently present antigens on MHCI and MHCII is their reduced ability for endosomal degradation. Although proteolysis is essential to the generation of MHC peptides, too much proteolytic activity leads to complete protein degradation into amino acids. Indeed, DCs are distinguished from other phagocytic cells (e.g., macrophages) by a remarkably low expression level of lysosomal proteases and a high lysosomal pH (92–94). The antigen susceptibility to degradation even by these reduced levels of proteases is a determinant factor to the efficiency at which MHCII–peptide complexes can be generated (95). Studies performed with murine DCs suggest that the MHCI pathway may be even more sensitive to lysosomal degradation. Indeed, inhibition of lysosomal proteases promotes antigen cross-presentation (96, 97). Murine CD11b^−^ CD8α^+^ DCs, which exhibit an increased ability for cross-presentation in comparison to the CD11b^+^ CD8α^−^ DCs, also generate high levels of reactive oxygen species in a NOX-2-dependent fashion so that their endocytic compartments stay at a more alkaline pH, thereby limiting antigen destruction (98). In addition, this phenomenon may also act to weaken or disrupt the vesicular membrane (99). As a result, antigen transport in the cytosol is increased. In addition, CD11b^−^ CD8α^+^ DCs also have higher levels of lysosomal inhibitors and lower levels of lysosomal proteases than CD11b^+^ CD8α^−^ DCs (46, 100). The constitutive activation of IRE-1α, a sensor of ER stress, is also a unique feature of CD11b^−^ CD8α^+^ DCs and appears essential to antigen cross-presentation (101). The precise mechanism by which activated IRE-1α promotes the MHCI cross-presentation pathway remains to be elucidated. At least, some of the features of the murine CD11b^−^ CD8α^+^ DCs are shared by human tonsil resident BDCA3^+^ DCs but also BDCA1^+^ DCs, both of which display similar cross-presentation capacity (51). Additionally, the three DC subsets efficiently export internalized proteins to the cytosol. However, another study found that blood BDCA3^+^ DCs superior at cross-presenting antigen delivered to lysosomes (28). Furthermore, blood BDCA3^+^ DCs express lower levels of lysosomal proteases than BDCA1^+^ DCs, suggesting that perhaps enhanced antigen release into the cytosol is favored by reduced lysosomal degradation. The lysosomal pH of blood DCs was not measured, and in the aforementioned study intracellular targeting of the antigen was not characterized. Further analysis will be needed to determine if different BDCA3^+^ DC subsets display different properties.

Finally, recent studies from our group and others suggest that both early and late endosomal compartments are capable of serving as antigen portals for cytosolic entry and cross-presentation. However, early endosomal compartments appear to be far more efficient for some antigens. This is not dependent on internalization levels, but rather the low proteolytic activity of early endosomes (28, 79, 80, 97, 102). Surprisingly, there does not seem to be a direct correlation between the level of internalization and cross-presentation. CD40 and mannose receptor/CD206 both deliver antigen to early endosomes, but CD40, the receptor that is the least efficiently internalized, turns out to be the most efficient at promoting cross-presentation (79). Slow antigen internalization might preserve antigen and provide a continuous “time-release” pool of antigen that might be used over extended periods for the continuous formation of peptide–MHCI complexes. The importance of targeting antigen to compartments with low proteolytic activity most likely depends on the nature antigen and its stability. Chatterjee et al. used long peptides as antigen which are particularly susceptible to degradation and probably have reduced ability to survive long enough to escape into the cytosol. Protein antigens, however, may be inherently more resistant. This could explain why in some systems antigen delivered to lysosomes using DEC205 or FcγR, are efficiently cross-presented, with similar or better efficacy as antigen delivered to early endosomes via mannose receptor/CD206 (103–105).

Collectively, the data reviewed in this section indicate that targeting receptors for antigen delivery to DCs can promote CD8^+^ T-cell responses by increasing the amount of antigen delivered to the desired DC subset(s). It can also enhance antigen presentation by controlling its intracellular routing and degradation, and extend antigen cross-presentation to DCs that might not be optimally equipped.

## Adjuvant

In absence of stimulation at steady-state DCs can induce tolerance. Antigen inoculation in absence of adjuvant leads to T-cell anergy or T-cell deletion (17, 72), and can induce regulatory T cells in the periphery (106–109). Hence, *in vivo* delivery of antigens to DCs in absence of adjuvant may also be a promising strategy to treat autoimmune disorders as reviewed elsewhere (110). But, to induce immunity rather than tolerance, it is essential to provide the DCs with an activation signal or “adjuvant” in addition to the vaccine antigen. Conserved components of microorganisms, or pathogen-associated molecular patterns (PAMPs) have been best characterized for their ability to activate DCs and their discovery offers the prospect of developing new vaccine adjuvants. PAMPs are recognized by pattern recognition receptors (PRRs) of the innate immune system. PRRs comprise a variety of receptors, including TLRs, cytosolic receptors [nucleotide-binding oligomerization domain-like (NOD-like) receptors (NLRs), RIG-I-like receptors (RLRs)], and C-type lectin receptors (111, 112). Activation of PPR signaling in DCs results in the enhancement of antigen presentation on MHCI and MHCII, cytokine production, and the upregulation of costimulatory molecules that are necessary for the induction of T-cell responses (5). Importantly, the nature of the adjuvant determines the type, the magnitude, the breadth, and the quality of the adaptive immune response. Differential patterns of expression of PRRs among DC subsets and different cytokine profiles induced by the triggering of distinct PRRs account for much of the diversity of phenotypes of the immune response (111, 113, 114) (Figure 1). Adding yet another level of complexity, adjuvants that trigger different pathways within a cell (115–117), or stimulate multiple cell types can cooperate to further enhance immune responses (70, 114, 118). In addition to PPRs, it was recently found that induction of stress response through sensing of amino acid starvation in DCs initiates autophagy and enhances MHCI cross-presentation (119). Stress sensors could therefore be possibly targeted to potentiate adjuvants.

The use of the mouse model to study and select adjuvants for human vaccine is limited because the pattern of expression of PRR can significantly differ between the two species. Because non-human primates express a similar repertoire of TLRs on immune cells to humans, they are a more relevant model to evaluate adjuvant effects (120, 121). While most adjuvants can induce antibody responses, generation of CD8^+^ T-cell immunity has proved particularly difficult (122). Immunization studies in non-human primates showed that Poly ICLC which stimulate multiple PPRs (TLR3, RIG-I, and MDA-5) and TLR7/8 agonists are currently the most potent known adjuvants for induction of T helper 1 and CD8^+^ T-cell responses (123–126). Poly ICLC and TLR7/8 agonist are the only TLR ligands capable of inducing both IL-12 and type I interferon, which are required for efficient cross-priming (53, 70, 114, 118). In mice, multiple cell types need to be stimulated for the production of IL-12 and type I interferon. IL-12 is produced by mDCs in response to Poly ICLC (through TLR3 triggering) and TLR7/8 agonist stimulation, whereas type I IFN is largely produced non-hematopoietic cells in response to Poly ICLC stimulation through MDA-5, and pDCs in response to TLR7/8 agonist, respectively. However, in mice reconstituted with a human immune system IL-12p70 and type I IFN production after TLR3 ligand stimulation resulted mainly from BDCA3^+^ DCs (53). Even more surprising is that those BDCA3^+^ DCs produce similar amounts of type I interferon as pDCs. These results are conflicting with those obtained after *in vitro* stimulation of BDCA3^+^ DCs isolated from human blood and human tissues which produce only limited amount type I interferon (28, 41). Further studies will be needed to confirm this observation. Another potential benefit of those TLRs is that they appear broadly expressed on human mDC subsets (Figure 1), and therefore they can engage multiple DC subsets, which has been shown to improve T-cell responses (70). Multiple clinical studies have been initiated to evaluate Poly ICLC and TLR7/8 agonists as vaccine adjuvants which will help establish their potency in humans (www.clinicaltrials.gov).

The co-delivery of adjuvant and antigen to DCs is critical for the priming of the immune response. Co-delivery has been realized by coupling antigen to adjuvant (127–129), fusing antigen to protein adjuvant, or co-encapsulation in particles (130–132), and has lead to significant increase in the magnitude of the immune responses and a better quality immune response (127). This enhanced T-cell priming may result from multiple effects: increased antigen uptake, altered intracellular routing, increased stability of the TLR agonist. The adjuvant effect may be even better achieved if the adjuvant and the antigen co-localize in the same endosomal compartments, as TLRs control MHCII presentation only in the compartments in which they are present (133, 134). Another benefit of coupled vaccines may be the local retention of the adjuvant at the site of injection, and thus the reduction of their toxicity. Indeed, free TLR agonists rapidly leave the site of injection and induce systemic innate responses resulting in high levels of serum cytokines (114). A more direct and controlled approach to reduce unwanted systemic effects of TLR agonists is to engineer their targeted delivery to DCs, although it might affect adjuvant effectiveness if activation of bystander cells contributes to the immune response (70, 118). Delivery of poly ICLC and TLR7/8 agonists through DEC205 or CD209 enhances DC activation and CD8^+^ T-cell response in mice. Moreover, potent CD8^+^ T-cell responses can be achieved with doses of adjuvant that do not induce toxic high serum cytokine levels (132).

Receptors other than TLRs have been shown to trigger DC activation. They are attractive due to their stimulatory capacity and their endocytic capacity that offer the potential of using a single molecule to deliver both antigen and activation signal to DCs. Dectin-1, a receptor involved in anti-fungal immunity, is a syk-coupled C-type lectin receptor that stimulate DC through its ITAM-like domain (112). Antigen delivery to human monocyte-derived DCs and BDCA1^+^ DCs through Dectin-1 leads to enhanced MHCI cross-presentation and cell activation *in vitro* (135, 136). However, mouse immunization studies suggest that Dectin-1 may be more potent at priming CD4^+^ T-cell responses than CD8^+^ T-cell responses (137). A more promising receptor may be the CD40 receptor, which is expressed by all DC subsets. Not only does it efficiently deliver antigen to the MHC presentation pathways in DCs (28, 79), but its ligation induces DC stimulation and promotes cross-presentation (138, 139). Immunization studies confirmed that anti-CD40 agonistic antibody/Ag conjugates can prime CD8^+^ T-cell responses in mice (140, 141). However, the use of agonist anti-CD40 antibodies in vaccine formulation may be limited by a narrow therapeutic window. CD40 is broadly expressed on B cells, monocytes, platelets, and endothelial cells, and CD40 ligation can induce high serum cytokine levels (142). It will be important to compare anti-CD40 antibodies with different agonistic function. Anti-CD40 with weaker agonistic function may be better tolerated and therefore allow higher antigen payload and vice versa for strong agonists. How this will impact the outcome of the immune response remains to be determined. CD32/FcγRII cross-linking also induces DC maturation and efficient antigen cross-presentation after immune complex internalization (73, 105, 143). Like CD40, it has the advantage of targeting most DCs, but could induce some toxicity because of its broad expression on other cells.

## Conclusion

Recent advances in DC biology and the mechanisms controlling adaptive immune responses have offered new insights for the rational design of novel vaccines. Immunization studies in mice indicate that there is a clear benefit to the targeting of antigens to DCs. A major challenge, however, remains to translate this approach developed in mice to humans. The preliminary data obtained from the first clinical trials testing vaccines targeting DEC205 (CDX-1401, Celldex) and mannose receptor/CD206 (CDX-1307, Celldex) indicate that this strategy can elicit immune responses (18–20), but maybe not as strong as one could have expected based on the mouse data. One explanation is that immunologist’s favorite model antigen for mouse studies is ovalbumin, which is exceptionally immunogenic, and may lead to overestimating vaccine efficacy. Mouse and human immune systems have also significant differences that make translation difficult (144). Although the intracellular mechanisms involved in antigen cross-presentation pathway and the DC lineage appear conserved between the two species, the specialization of the DC subsets may not be conserved. In addition, the pattern of expression of endocytic receptors for antigen delivery and TLRs for DC activation are different between mice and humans. Clearly, using a different model such as mice with a reconstituted immune system or non-human primates, which have a human immune system more similar to the human immune system is essential to optimize these vaccines. Additionally, analysis of the immune response to successful human viral vaccines that induce potent CD8^+^ T-cell responses could help further determine the mechanisms that control immune responses to vaccination and identify predictors of vaccine efficacy (145).

Another challenge specific to the therapeutic treatment of cancer and maybe persistent viral infection is that they developed mechanisms to evade immune clearance by impairing T-cell function (146). The presence of these suppressive factors may limit vaccine efficacy, and combination of a vaccine with immunomodulatory molecules to neutralize inhibitory signals may be necessary to produce effective T-cell immune response.

In spite of these challenges, we view the present as an exciting time to study vaccine development and foresee that continuing to design DC-based therapies will allow us to prevent and treat many of the major illnesses for which no vaccine currently exists.

## Conflict of Interest Statement

Lélia Delamarre is an employee of Genentech, and hence declares a competing financial interest. Lillian Cohn declares no conflict of interest.
